# Effect of a short-term exercise program on glycemic control measured by fructosamine test in type 2 diabetes patients

**DOI:** 10.1186/1758-5996-6-16

**Published:** 2014-02-11

**Authors:** Bruno P Moura, Paulo RS Amorim, Bruno PP Silva, Sylvia CC Franceschini, Janice S Reis, João CB Marins

**Affiliations:** 1 Department of Physical Education, Human Performance Laboratory, Universidade Federal de Viçosa, Viçosa, Minas Gerais, Brazil; 2 Department of Nutrition, Universidade Federal de Viçosa, Viçosa, Minas Gerais, Brazil; 3 Institute of Education and Research of Santa Casa de Belo Horizonte, IER-SCBH, Belo Horizonte, Minas Gerais, Brazil

**Keywords:** Diabetes management, Physical exercise, Glucose assessment, Aerobic exercise, Cycle ergometers, Cardiorespiratory fitness

## Abstract

**Background:**

Glycated hemoglobin (A1C) and Fasting Plasma Glucose (FPG) are the two monitoring blood glucose tests most frequently used. However, both methods are shown to be insensitive to detect glycemic variations in short duration periods. Therefore, we aimed to assess the effect of a short-term exercise program on glycemic levels measured by fructosamine concentrations in type 2 diabetes patients.

**Methods:**

Eight volunteers (51.1 ± 8.2 years) underwent a supervised exercise program during eight weeks (3 d.wk^-1^, 50-60% of VO_2_ peak for 30–60 minutes). The body composition, VO_2_ peak, A1C, FPG, fructosamine and capillary blood glucose (CBG) were evaluated. We used ANOVA - One Way for repeated measures followed by Tukey post-hoc test and paired t test. P values <0.05 were considered significant.

**Results:**

We found statistical differences on the concentrations of fructosamine, VO_2_ peak and CBG. However, A1C and FPG showed no statistical difference. Fructosamine declined by 15% (57 μmol/L) between the beginning and the end of the study. Individually, 50% of the sample reached the reference values for the normality in fructosamine test. VO_2_ peak increased by 14.8% (3.8 ml.kg^-1^.min^-1^) and CBG decreased on an average of 34.4% (69.3 mg/dL).

**Conclusions:**

Fructosamine test is effective in the evaluation of glucose with type 2 diabetes patients when undergoing a short exercise program, alternatively to the traditional A1C and FPG assessment. Our results are relevant in clinical practice, because the significant improvement in glycemic status can help to evaluate the inclusion of exercise as adjunct therapy to replace the prescription of additional drugs in poorly controlled patients.

## Background

Glycemic control is a major therapeutic goal for type 2 diabetes patients. The deleterious effects of glucotoxicity have an important role in the progressive impairment of insulin secretion and sensitivity, two major factors in the pathogenesis of type 2 diabetes, which can lead to future microvascular diseases or others complications [1]. Studies have shown that glycemic control is the key to diabetes control, because it is associated with significant reductions of microvascular complication rates (retinopathy and nephropathy) and neuropathy [2].

One way to achieve a good glycemic control is through exercise [3] that has a well-known effect on type 2 diabetes [4]. Thus exercise has been recommended for diabetics because increase glucose uptake to active muscle and decrease the risk of cardiovascular diseases [5,6].

Glycated hemoglobin (A1C) and Fasting Plasma Glucose (FPG) are the most common analysis for monitoring blood glucose levels [2]. However, A1C reflects a glycemic control over a period of 2–3 months, which is too long to assess the effectiveness of short-term exercise programs on glycemic control [7]. Likewise, the FPG is very variable because is influenced by the glycemic content ingested in the diet from the previous night [8].

Fructosamine (measure of glycated proteins, specifically albumin) has a half-life shorter than the red cell. Therefore the fructosamine is presented as an indicative of glycemic control on period of 2–3 weeks [9]. Thus, this test is particularly useful when assessing periods of rapid change in glycemia [10], such as those resulting from a short-term exercise programs [9,11,12].

Therefore, we aimed to assess the effect of a short-term exercise program on glycemic levels measured by fructosamine concentrations in type 2 diabetes patients.

## Methods

### Patients

Patients recruitment for this study occurred through advertisements in local media, which were selected according to the following inclusion criteria: a) Have type 2 diabetes diagnosed by criteria of the American Diabetes Association (ADA) [2]; b) Must not have diabetes complications (cardiovascular disease, neuropathy, retinopathy and nephropathy); c) Must not have practiced physical exercises with professional guidance in the last 2 months preceding the start of the program; d) Agreed not to do other kinds of physical exercises with professional guidance in addition to those on the program during the study and maintain their usual diets throughout the intervention period.

Twenty volunteers signed up to participate in the study, fifteen of which were approved by inclusion criteria. After a meeting to explain all the study procedures, eleven patients confirmed their participation. After the first week of the study, one was excluded due to an impediment in performing physical exercise because of ulceration on the feet. Two other gave up during the exercise program for personal reasons. Finally, eight patients (average age 51.1 ± 8.2 years, diagnosis: 5.1 ± 4.6 years ago), four of whom were women, composed the final sample. All patients were on use of medications since diagnosis of diabetes (metformin, insulin, and glimepiride). No patient reported changing in his dose of medication during the study. Though, recent study showed that metformin did not significantly attenuate the benefits of exercise on glycemic control or fitness [13].

All patients enrolled in this study had the approval of their physician to practice physical exercise of moderate intensity and signed terms of consents. The study was approved by the Ethics Committee on Human Research of Universidade Federal de Viçosa.

### Anthropometry and body composition assessment

Anthropometric measurements were obtained at the Human Performance Laboratory (LAPEH), by a trained examiner. Weight, height and waist circumference measurements were performed by calibrated equipment based on the procedures described by Lohman et al. [14]. Body mass index (BMI) was calculated and analyzed according to guidelines of the World Health Organization (WHO) [15]. Body composition assessment was performed by the apparatus Body Composition Analyser (BIA 310 bioimpedance analyzer, Biodynamics Corp.) [16].

### Biochemical tests

Blood sample collection was obtained from the median cubital vein after eight hours fasting by a trained biochemist. Technique of vacuum collection tubes with EDTA-K_3_ was used for hematology (A1C and Fasting Plasma Glucose) and tubes with clot accelerator (SiO_2_) and gel separator was used for serology (Fructosamine). The samples were analyzed in the Clinical Laboratory, Division of Health, at Universidade Federal de Viçosa. The following methods were used for analysis: a) Glycated Haemoglobin (A1C): HPLC (High Performance Liquid Chromatography), by the device VARIANT II System (Bio-Rad Laboratories, Inc., USA) with reference value (RV) for normality ≤ 6.5% [2]; b) Fructosamine: colorimetric with reduction of nitroblue tetrazolium (NBT) by device Modular (Roche), with RV for normal 205–285 μmol/L and c) Fasting Plasma Glucose (FPG): glucose oxidase method in device Cobas Mira Plus (Roche), with RV for diabetes ≥ 126 mg/dL (7.0 μmol/L) [2]. Capillary Blood Glucose (CBG) was measured, before and after each exercise session, by blood glucose monitor Accu-Chek Go (Roche).

### Cardiorespiratory fitness

Cardiorespiratory fitness of patients was measured by the metabolic gas analyzer VO2000 (Medical Graphics Corporation) and analyzed by software Aerograph 4.3 (Medical Graphics Corporation). Metabolic gas analyzer was automatic calibrated before each test. Cardiorespiratory fitness tests were performed on a cycle ergometer (ISO1000, SCIFIT® Corporate Headquarters). All necessary precautions for to perform cardiorespiratory fitness tests in diabetic patients (e.g. blood pressure measurement at rest and during testing, measurement of blood glucose before and after the test) was taken. Prior to cardiorespiratory fitness tests, patients performed an adaptation to cycle ergometer through a warm-up five minutes. Ramp protocol was used with increments to charge by the minute; in which patients were encouraged to reach 85% of heart rate (HR) estimated by HR_max._ = 208 – (age × 0.7) proposed by Tanaka et al. [17]. Because we do not have the presence of a physician during the test of cardiorespiratory fitness, for security reasons, when 85% of maximal HR was reached, the cardiorespiratory fitness test was interrupted and started the recovery stage. Test data (HR and VO_2_) were used as a basis for the development of individual equations to estimate peak oxygen consumption (VO_2_ peak) by linear regression (SigmaPlot® Version 11.0; Systat Software, Inc., Chicago, IL, USA) [18].

### Protocol

**Figure 1:**
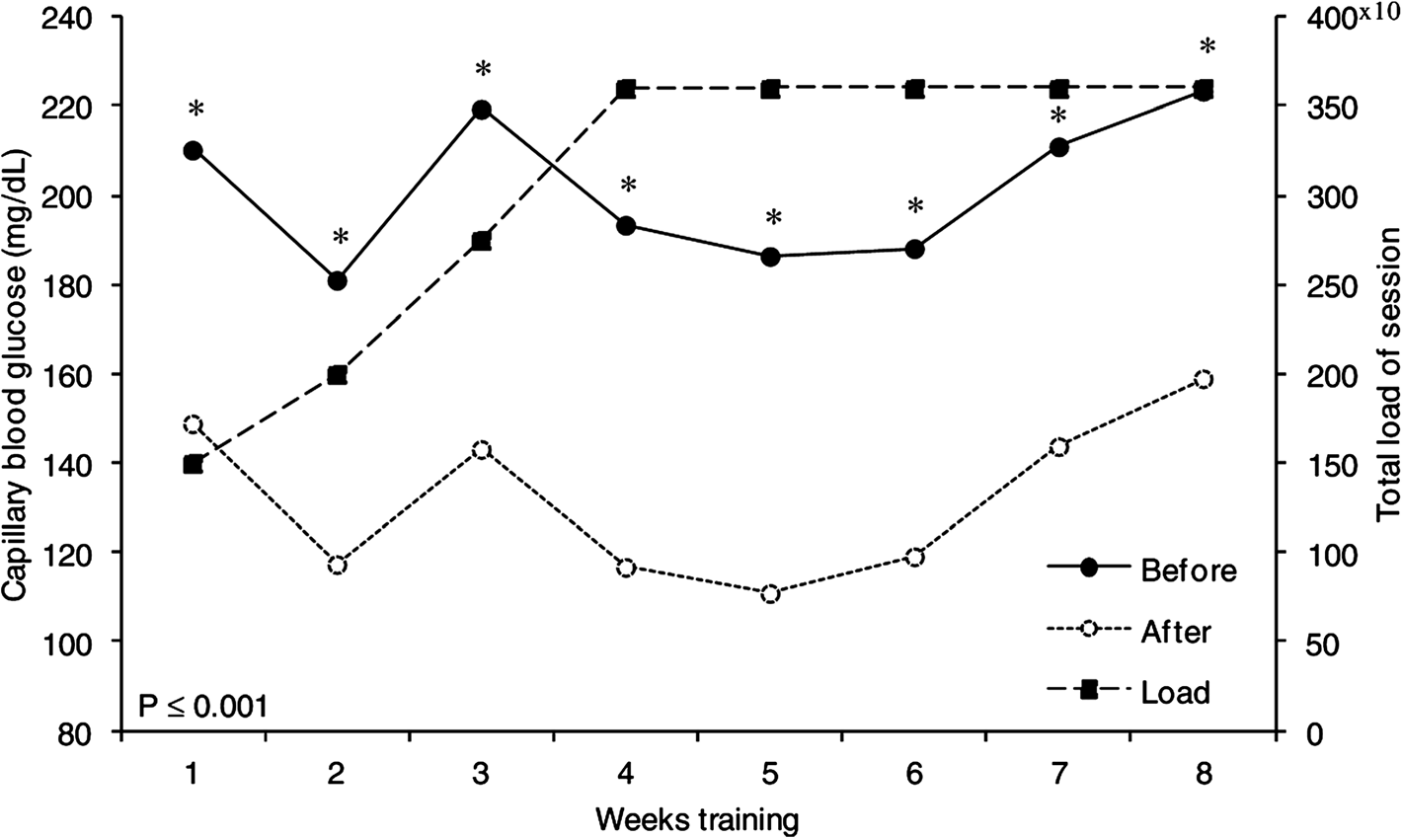
Average weekly blood glucose before and after exercise sessions and the evolution of training load.

The study lasted ten weeks, in which eight weeks were intervention with physical exercise (Table 1). The exercises were performed into Human Performance Laboratory, at Universidade Federal de Viçosa, by cycle ergometers (Cycle 167, 2001, ERGO-FIT®). Physical exercises were performed three days per week (d.wk^-1^) over eight weeks. Throughout the study, duration bout ranged from 30 to 60 minutes and intensity ranges from 50% to 60% of VO_2_ peak (Table 1). Training bout was divided into three phases: warm-up, physical exercise and cool down. The warm-up and cool down always lasted five minutes each. Intensity control based on VO_2_ peak was performed by monitoring the corresponding HR, through the HR monitor (Polar® RS800CX, Finland). Patients were asked to maintain an average speed of 20 km/h throughout the exercise and the load (watts) on the cycle ergometer was increased up to achieve the target HR based on the percentage of VO_2_ peak for the specific bout. However, if a participant presented peripheral fatigue, the load (watts) on the cycle ergometer was reduced and the exercise speed has increased, in order to maintain physiological load (HR) required. The total load of the session (Figure 1) was calculated by the following equation: Total load of session = Intensity × Duration [19]. Fructosamine analysis was performed pre, during and post study (Weeks 1, 5 and 10) (Table 1). We do not control food intake of the patients and they were asked to maintain their usual diet throughout the physical exercise period.

**Table 1:** Drawing of the progression of the study

**Weeks**	**1**^**Ev**^	**2**	**3**	**4**	**5**^**Fru**^	**6**	**7**	**8**	**9**	**10**^**Ev**^
Exercise period	Exercise									
Intensities		50	50	55	60	60	60	60	60	
Duration/session		30	40	50	60	60	60	60	60	

Legend: Ev = Evaluation (Anthropometry and body composition, biochemical and cardiorespiratory fitness tests); Fru = Fructosamie examination during exercise intervention); Intensities = percentage of VO_2_ peak and Duration/Session = Minutes.

### Statistics

All variables were approved by normality test (Shapiro-Wilk) and Equal Variance test. Thus, results are presented as mean ($\bar{x}$) and standard deviation (SD). ANOVA - One Way for repeated measures followed by Tukey post-hoc test was used to assess the evolution of fructosamine during the study. For the other variables, paired t test was used to compare the performance pre and post intervention, and pre and post exercise session for the capillary blood glucose. P values <0.05 were considered significant. The tests were performed by the software SigmaPlot Version 11.0 (Systat Software, Inc., Chicago, IL, USA).

## Results

**Figure 2:**
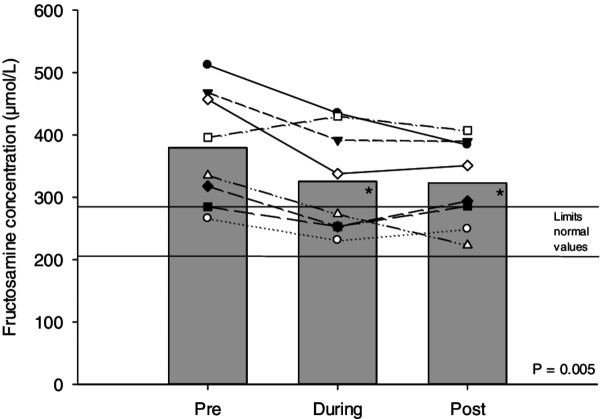
Mean concentration of fructosamine pre, during and post study.

We found significant statistical differences on fructosamine concentrations between the study periods (P values = 0.05; Power of performed test = 0.85 – Figure 2), VO_2_ peak (Table 2) and CBG (Figure 1) before and after exercise sessions.

**Table 2:** **Anthropometric variables, body composition, A1C, FPG and VO**_**2 **_**peak of subjects pre (n = 8) and post (n = 8) study**

	**Pre**	**Post**	
**Variables**	$\bar{x}$ **± SD**	$\bar{x}$ **± SD**	**P values**
**Weight (kg)**	83.7 ± 17.1	83.2 ± 17.7	0.195
**BMI (kg/m**^**2**^**)**	30.2 ± 6.4	29.8 ± 6.2	0.063
**Waist circumference (cm)**	102.6 ± 12.0	97.5 ± 20.1	0.312
**% Fat**	33.4 ± 6.3	32.4 ± 5.9	0.245
**Fat mass (kg)**	28.6 ± 9.8	27.4 ± 9.3	0.137
**A1C (%)**	8.9 ± 1.6	8.3 ± 1.8	0.083
**FPG (mg/dL)**	192.2 ± 73.9	176.7 ± 55.1	0.459
**Peak VO**_**2 **_**(ml.kg.min**^**-1**^**)**	25.7 ± 5.1	29.5 ± 6.6	0.048

Legend: BMI = Body Mass Index; % Fat. = Body Fat Percentage; A1C = Glycated hemoglobin e FPG = Fasting Plasma Glucose. Data are showed as mean ($\bar{x}$) ± Standard Deviation (SD).

Fructosamine test showed a decrease in blood concentration of 15% (57 μmol/L) between the beginning and the end of the study, while VO_2_ peak showed an increase of 14.8% (3.8 ml.kg^-1^.min^-1^). Capillary blood glucose during exercise sessions showed an average decrease of 34.4% (69.3 mg/dL).

Anthropometric variables, body composition, A1C and FPG showed no statistical difference between pre and post study periods (Table 2). However, when assessing the BMI, according to World Health Organization (WHO) [15], the classification of the subjects were in the state of obesity class I, confirmed by the high percentage of body fat measured by bio impedance technique.

## Discussion

Evidences from this study support the use of fructosamine test to assess the effect of a short-term exercise program on glycemic control in type 2 diabetes patients. This test showed a decrease of 14.2% from the first to fifth week of the study (after three exercise weeks) and 0.8% from the fifth to tenth week (Figure 2).

Individually, 50% of the sample reached RV. Two subjects reached the RV in the fifth week and remained in that range until the end of the study, while two others reached the RV in the fifth week but failed to keep them until the end of the study. These results are relevant in clinical practice, because the significant improvement of 15% in glycemic status after eight weeks of physical exercise can help to evaluate the exercise inclusion as adjunct therapy to replace the prescription of additional drugs in poorly controlled patients.

Studies has recommended the use of fructosamine test for glycemic control in short periods of monitoring, which may occur fast changes of blood glucose levels [7,9,20-24]. Raz et al. [12] investigated the influence of 12 weeks moderate exercise on the parameters of glycemic control in type 2 diabetes patients and found a significant reduction in the fructosamine levels. Unlike the studies cited above, the current manuscript assessed the effect of short-term exercise program on glycemic control measured by fructosamine test. Taken together, results from these studies provide evidence for the use of fructosamine test for glycemic control short-term exercise program.

Rychlewski e Szczesniak [11] investigated the effects of regular exercise along three weeks on fructosamine levels in children with type 1 diabetes. They found a reduction in the frutosamine levels, as well as increased insulin sensitivity. Although the results presented above involve patients with type 1 diabetes, they are quite similar to the ones presented in the current study, involving type 2 diabetes patients. However, the improvement of glycemic control observed could likely be maximized with the continuation of the exercise program in conjunction with new increments in the training loads.

The importance of fructosamine test as the method of measuring glycemic status during short-term exercise program is most apparent when viewed in conjunction with the evaluation of A1C and FPG (Table 2). Both tests often used as a way of controlling the glycemic levels, despite showing a reduction that can be considered clinically important, showed no statistically significant differences before and after study. The traditional tests (A1C and FPG) used in the assessment of glycemic control showed a lower sensitivity to indicate the beneficial changes induced by exercise, as assessed by fructosamine test. This fact may explain unsatisfactory results of the effect of a short-term exercise program on glycemic control sometimes found in the literature [25,26].

In a study conducted in a developing country, the use of fructosamine test presented itself as a good alternative for the A1C test [27]. This test may be particularly appropriate for developing countries like Brazil, where the supply of strips for blood glucose control is not accessible to type 2 diabetes patients that are not being insulinized.

After the fourth exercise week, the training load remained unaffected (Figure 1). Because of a difficulty in generating incremental load due to the fact that diabetic patients has a reduced fitness [5]. However, even with the maintenance of training load, the reduction in CBG after weekly exercise sessions remained statistically significant (P ≤ 0.001) until the end of the exercise program.

Evolution of training load in relation to fructosamine test results, it appears that fructosamine concentration (Figure 2) remained practically unchanged after the stabilization of the workload. This fact can be explained by the physiological adaptations triggered by the exercise in the body and shows need of workload increases to ensure the continuity of the beneficial effects of exercise on glycemic control. However, even with the difficulty of increasing training loads, after the fourth exercise week, the subjects showed an improvement in cardiorespiratory fitness (Table 2). These results confirm that the intensity used in the exercise program was adequate to cause physiological adaptations.

The lack of alteration of anthropometric variables and body composition presented in the current study are corroborated by the study of Boulé et al. [28]. These authors conducted a meta-analysis about exercise effects on glycemic control and body mass in type 2 diabetes patients. They found that exercise effects on glycemic control are independent of weight loss.

The main limitations of this study were the small sample size and the absence of a control group. These facts occurred due to the difficulty in finding volunteers with type 2 diabetes without microvascular diseases or others complication, able and willing to participate in a program of controlled exercise for eight weeks. However, all variables were approved by the normality test and present parametric distribution. Another limitation was the lack of control over food intake, despite recommendations to maintain their usual diets are continually enforced on all subjects throughout the intervention period.

Thus, addition to sensitivity in detecting the effects of a short-term exercise program on glycemic control, fructosamine test has additional advantages. Such as realization in any day time without the need to fasting, low cost, as well as the fact that there are available domestic monitoring devices with the validity and accuracy evidenced in relation to laboratory tests [29]. A key aspect to type 2 diabetes patients that should be highlighted is the existence of studies linking the reduced fructosamine levels with decreased cardiovascular risk and mortality rates [10,24].

## Conclusions

The results of our study support the use of fructosamine test to assess the effect of a short-term exercise program on glycemic control in type 2 diabetes patients. This test showed as a good alternative compared to traditional measures of glycemic control (A1C and FPG).

Our results are relevant in clinical practice, because the significant improvement in glycemic status after eight weeks of physical exercise can help to evaluate the inclusion of exercise as adjunct therapy to replace the prescription of additional drugs in poorly controlled patients.

Nevertheless, further studies with a larger sample, diet control and exercise programs with constant workloads increments should be performed to replicate the results verified in our study.

## Competing interests

The authors declare that they have no competing interests.

## Authors’ contributions

BPM and PRSA were responsible for the study design. BPPS was responsible for overseeing the exercise training of volunteers. JCBM, SCCF and JSR were co-advisors and made corrections in the text. All authors read and approved the final paper.

## References

[B1] Giaccari A Sorice G Muscogiuri G Glucose toxicity: the leading actor in the pathogenesis and clinical history of type 2 diabetes - mechanisms and potentials for treatment Nutr Metab Cardiovasc Dis 2009 19 365 377 10.1016/j.numecd.2009.03.018 19428228

[B2] American Diabetes Association Standards of medical care in diabetes--2011 Diabetes Care 2011 34 Suppl 1 S11 S61 21193625 10.2337/dc11-S011 PMC3006050

[B3] Moura BP Natali AJ Marins JCB Amorim PRS Different approaches of physical training used in the management of type 2 diabetes: a brief systematic review of randomised clinical trials Brit J Diabetes Vasc Dis 2011 11 210 216 10.1177/1474651411410578

[B4] Colberg SR Sigal RJ Fernhall B Regensteiner JG Blissmer BJ Rubin RR Chasan-Taber L Albright AL Braun B Exercise and type 2 diabetes: the American College of Sports Medicine and the American Diabetes Association: joint position statement Diabetes Care 2010 33 e147 e167 10.2337/dc10-9990 21115758 PMC2992225

[B5] McGavock JM Eves ND Mandic S Glenn NM Quinney HA Haykowsky MJ The role of exercise in the treatment of cardiovascular disease associated with type 2 diabetes mellitus Sports Med 2004 34 27 48 10.2165/00007256-200434010-00004 14715038

[B6] Marwick TH Hordern MD Miller T Chyun DA Bertoni AG Blumenthal RS Philippides G Rocchini A Exercise training for type 2 diabetes mellitus: impact on cardiovascular risk: a scientific statement from the American Heart Association Circulation 2009 119 3244 3262 10.1161/CIRCULATIONAHA.109.192521 19506108

[B7] Hom F Fructosamine, hemoglobin A1c, and measures of diabetic control Diabetes Technol Ther 1999 1 443 445 10.1089/152091599316973 11474830

[B8] Liu K Stamler J Stamler R Cooper R Shekelle RB Schoenberger JA Berkson DM Lindberg HA Marquardt J Stevens E Tokich T Methodological problems in characterizing an individual’s plasma glucose level J Chronic Dis 1982 35 475 485 10.1016/0021-9681(82)90062-5 7076788

[B9] Klonoff DC Serum fructosamine as a screening test for type 2 diabetes Diabetes Technol Ther 2000 2 537 539 10.1089/15209150050501943 11469616

[B10] Misciagna G De Michele G Trevisan M Non enzymatic glycated proteins in the blood and cardiovascular disease Curr Pharm Des 2007 13 3688 3695 10.2174/138161207783018545 18220807

[B11] Rychlewski T Szczesniak L [Fructosamine in blood serum, binding and degradation of 125J-insulin by erythrocyte receptors in young persons with type I diabetes--effect of physical exercise] Pol Arch Med Wewn 1996 95 212 217 8755851

[B12] Raz I Hauser E Bursztyn M Moderate exercise improves glucose metabolism in uncontrolled elderly patients with non-insulin-dependent diabetes mellitus Isr J Med Sci 1994 30 766 770 7960690

[B13] Boule NG Kenny GP Larose J Khandwala F Kuzik N Sigal RJ Does metformin modify the effect on glycaemic control of aerobic exercise, resistance exercise or both? Diabetologia 2013 56 11 2378 2382 10.1007/s00125-013-3026-6 23975325

[B14] Lohman TG Roche AF Martorell R Anthropometric standardization reference manual 1988 Champaign, IL: Human Kinects

[B15] WHO World Health Organization. Diet, nutrition and the prevention of chronic diseases World Health Organ Tech Rep Ser 2003 916 1 149 12768890

[B16] Lukaski HC Bolonchuk WW Hall CB Siders WA Validation of tetrapolar bioelectrical impedance method to assess human body composition J Appl Physiol 1986 60 1327 1332 3700310 10.1152/jappl.1986.60.4.1327

[B17] Tanaka H Monahan KD Seals DR Age-predicted maximal heart rate revisited J Am Coll Cardiol 2001 37 153 156 10.1016/S0735-1097(00)01054-8 11153730

[B18] Ekelund U Franks PW Sharp S Brage S Wareham NJ Increase in physical activity energy expenditure is associated with reduced metabolic risk independent of change in fatness and fitness Diabetes Care 2007 30 2101 2106 10.2337/dc07-0719 17536069

[B19] Caballero JAR Manso JMG Valdivielso MN Caballero JAR Bases teoricas del entrenamiento deportivo 1996 1 Madrid: S.L. Editorial Gymnos

[B20] Post EM Moore JD Ihrke J Aisenberg J Fructosamine levels demonstrate improved glycemic control for some children attending a diabetes summer camp Pediatr Diabetes 2000 1 204 208 10.1046/j.1399543X.2000.010406.x 15016217

[B21] Gomo Z Serum fructosamine: a parameter for monitoring metabolic control in diabetes Cent Afr J Med 1992 38 358 362 1298563

[B22] Yahaya IA Isah HS Anaja PO Serum fructosamine in the assessment of glycaemic status in patients with sickle cell anaemia Niger Postgrad Med J 2006 13 95 98 16794643

[B23] Lindsey CC Carter AW Mangum S Greene D Richardson A Brown SJ Essary JL McCandless B A prospective, randomized, multicentered controlled trial to compare the annual glycemic and quality outcomes of patients with diabetes mellitus monitored with weekly fructosamine testing versus usual care Diabetes Technol Ther 2004 6 370 377 10.1089/152091504774198070 15198841

[B24] Browner WS Pressman AR Lui LY Cummings SR Association between serum fructosamine and mortality in elderly women: the study of osteoporotic fractures Am J Epidemiol 1999 149 471 475 10.1093/oxfordjournals.aje.a009835 10067907

[B25] Snowling NJ Hopkins WG Effects of different modes of exercise training on glucose control and risk factors for complications in type 2 diabetic patients: a meta-analysis Diabetes Care 2006 29 2518 2527 10.2337/dc06-1317 17065697

[B26] Bello AI Owusu-Boakye E Adegoke BO Adjei DN Effects of aerobic exercise on selected physiological parameters and quality of life in patients with type 2 diabetes mellitus Int J Gen Med 2011 4 723 727 22114516 10.2147/IJGM.S16717 PMC3219758

[B27] Islam N Akhter J Kayani N Khan MA Fructosamine: an alternative assessment of past glycaemic control in developing countries J Pak Med Assoc 1993 43 238 240 8114262

[B28] Boule NG Haddad E Kenny GP Wells GA Sigal RJ Effects of exercise on glycemic control and body mass in type 2 diabetes mellitus: a meta-analysis of controlled clinical trials JAMA 2001 286 1218 1227 10.1001/jama.286.10.1218 11559268

[B29] Edelman SV Callahan P Deeb LC Multisite evaluation of a new diabetes self-test for glucose and glycated protein (fructosamine) Diabetes Technol Ther 2000 2 233 238 10.1089/15209150050025195 11469264

